# Angina Bullosa Hemorrhagica of the Oral Mucosa: A Case Report

**DOI:** 10.7759/cureus.92123

**Published:** 2025-09-12

**Authors:** Prarthana Sudeep, Krishna Santhosh Kumar, Aravind MS, Renju Jose

**Affiliations:** 1 Department of Oral Medicine and Radiology, Amrita School of Dentistry, Amrita Vishwa Vidyapeetham, Kochi, IND

**Keywords:** angina bullosa hemorrhagica, blood blisters, oral mucosal ulcers, soft palate, trauma

## Abstract

Angina bullosa hemorrhagica (ABH) is a rare and benign condition characterized by the abrupt emergence of one or more blisters filled with blood inside the oral cavity. These blood-filled vesicles and bullae, which appear on the palate or oropharynx, are acute and occasionally painful. Its etiopathogenesis is unclear; however, it cannot be linked to systemic illnesses, vesiculobullous disorders, blood dyscrasias, or any other recognized cause. They were also identified in patients who use topical steroids or have diabetes mellitus or hypertension. Usually, the blisters burst within a day or two and heal on their own without causing any more pain or scars. The lesions are typically isolated and may cause patient anxiety due to a sudden onset. This case report’s primary goal is to raise awareness to prevent misdiagnosis and improper treatment of the condition.

## Introduction

In clinical practice, oral mucosal lesions are frequently encountered, and they encompass a wide variety of presentations ranging from acute traumatic injuries to chronic ulcerative conditions. While many traumatic lesions heal uneventfully, some may present as blisters, erosions, or ulcerations that mimic other disease entities. Most of them are acute or long-term soft tissue injuries resulting from a variety of factors, including mechanical trauma, prosthesis-related irritation, and thermal injury. They only rarely develop into burns and post-traumatic mucosal sores, which are artefactual concerns. Their source, location, and clinical manifestations, however, may vary greatly [1]. Because of this, accurate identification is essential for avoiding misdiagnosis and unnecessary treatment [2,3].

Angina bullosa hemorrhagica (ABH) is one such rare disorder of the oral mucosa that is represented by recurring episodes of bullae and vesicles filled with blood in the oral cavity. This condition is not brought on by autoimmune blistering diseases, systemic disorders, or blood dyscrasia [4]. The exact etiology of this benign oral pathology remains unclear; however, it is thought to arise in susceptible individuals following traumatic events and is linked to systemic conditions such as hypertension, diabetes mellitus, drug-induced thrombocytopenia, and chronic use of steroid inhalers [3,5]. Blisters cure on their own without leaving scars. First described as “traumatic oral hemoplyctenosis” by Balina in 1933, ABH has since been recognized as a distinct clinicopathologic entity, though its prevalence remains poorly established [6]. A Brazilian retrospective study reported that among 12,727 oral and maxillofacial lesions, only 0.18% were diagnosed as ABH [7].

The diagnosis is mainly clinical. This rarity, along with its clinical overlap with other mucocutaneous conditions, highlights the importance of clinician awareness. Associated lesions tend to recover on their own in a matter of days without therapy. Conservative and supportive measures remain the mainstay of therapy, with antibacterial mouthwashes and nonsteroidal anti-inflammatory drugs (NSAIDs) sometimes used for symptomatic relief. Although anxiolytics have been mentioned in the literature, their use in the routine management of ABH is rare [8].

## Case presentation

A 50-year-old female patient was referred to the department of oral medicine and radiology from ENT with a history of palatal bleeding for two days. On initial ENT examination, bilateral white patches with a raised surface were noted on the hard palate at the presumed bleeding sites. The patient reported that bleeding started after consuming hot tea, following which she drank cold water to arrest it. She also complained of pain, burning sensations, irritation, and difficulty in swallowing and eating.

The patient was a non-smoker and non-alcoholic. She had a medical history of type 2 diabetes mellitus and systemic hypertension for the past 10-15 years, as well as bronchial asthma managed with inhalers. A positive family history of diabetes mellitus was reported on both paternal and maternal sides.

**Figure 1:**
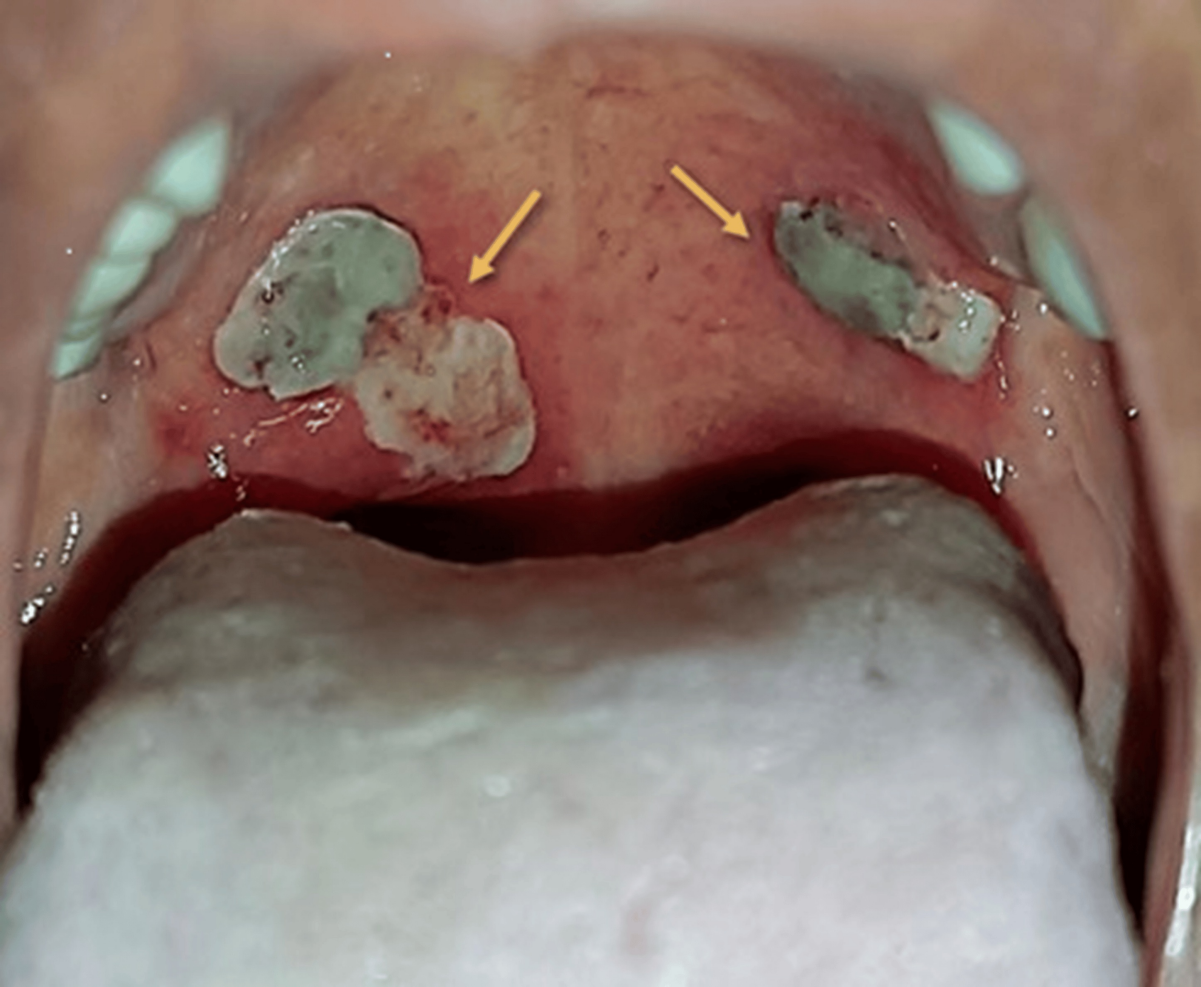
Clinical photograph showing bilateral ulcerated lesions on the soft palate (arrows) with a yellowish-white surface, surrounding erythematous halo, and irregular margins.

On further oral examination, ulcerated areas approximately 1.5 × 1 cm in size were observed on the soft palate bilaterally (two on the right side and one on the left) (Figure 1). The lesions had a yellowish-white surface with a mild blackish hue, surrounded by an erythematous halo and irregular borders. The adjacent mucosa appeared normal. On palpation, ulcers were tender and non-indurated, with no bleeding or discharge, and had an irregular surface.

The patient’s dentition was well-maintained with fair oral hygiene. No significant plaque deposits or periodontal pockets were observed.

Investigations

Routine blood investigations and a coagulation workup were advised. All vital signs were within normal limits. Random blood glucose (RBG) level was elevated. Bleeding and clotting times were within the normal range (Table 1). Unfortunately, glycosylated hemoglobin (HbA1c), prothrombin time (PT), and activated partial thromboplastin time (aPTT) were not performed, which has been acknowledged as a limitation. Advanced imaging modalities and biopsy were also not carried out. Based on history, clinical findings, and laboratory results, a provisional diagnosis of ABH was made.

**Table 1:** Summary of laboratory findings.

Laboratory test	Value/unit	Reference range
Random blood glucose	289 mg/dL	110-140 mg/dL
Bleeding time	2 minutes	Approximately 2-7 minutes
Clotting time	3 minutes	Approximately 8-15 minutes

Differential diagnosis

Other than the provisional diagnosis of ABH, differential diagnoses for the lesion included the following: (i) thermal burn, (ii) pemphigus, (iii) bullous pemphigoid, (iv) bullous lichen planus, (v) thrombocytopenia-associated ulcers, (vi) ulcers due to an infectious cause (viral or fungal), (vii) necrotizing sialometaplasia, and (viii) other benign inflammatory conditions of minor salivary glands.

Treatment

The patient was reassured, and symptomatic management was initiated for the oral lesions. The therapeutic regimen comprised prednisolone 0.5 mg tablets, to be swished and expectorated thrice daily for five days; benzydamine hydrochloride mouthwash, administered four times daily for five days; metronidazole gel, applied topically to the ulcers four times daily for five days; and clotrimazole 1% w/v oral paint, applied topically to the tongue and palate four times daily for five days.

Outcome and follow-up

**Figure 2:**
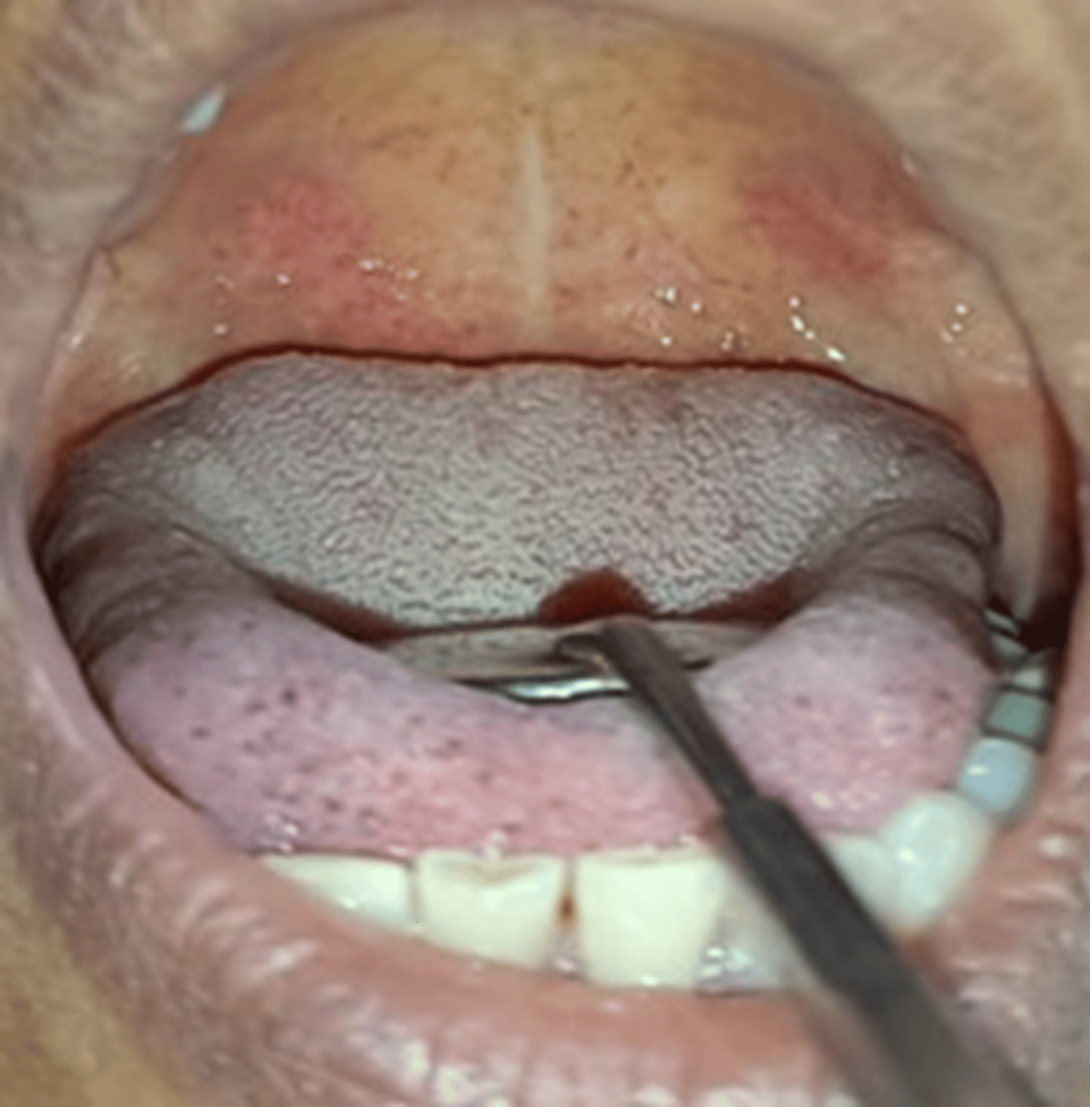
Follow-up clinical photograph after 10 days. Complete healing of the ulcerated palatal lesions was noted.

The follow-up after 10 days showed complete regression of the palatal lesions (Figure 2).

## Discussion

Badham first reported ABH as blood-filled blisters in the oral, pharyngeal, and esophageal mucosa in 1967 [9]. Although frequently described as rare, ABH is likely underrecognized due to its rapid onset and spontaneous resolution, and it is often misdiagnosed.

Numerous theories can be found in the literature; however, the etiology and pathology of ABH remain unclear [2]. High and Main discovered a connection between ABH and long-term inhaled corticosteroid usage [10]. Prolonged usage of corticosteroids can lead to epithelium atrophy and alter collagen synthesis [2,10]. According to Ordioni et al., 33% of patients with ABH had no prior medical history, whereas the incidence rates of inhaled corticosteroids, diabetes, and hypertension were 21%, 15%, and 7%, respectively, in patients with a medical history [2]. Based on current evidence, no definitive causal association with diabetes, hypertension, or corticosteroid use has been established. In the present case, the patient had long-standing diabetes and hypertension and reported regular use of inhalers for asthma. The precipitating factor appeared to be the consumption of hot tea, which aligns with the literature identifying mastication-related trauma and thermal stimuli as common triggers. The soft palate, as in this case, is the most frequently affected site due to its delicate mucosa and reflex vasodilation during mastication. Subepithelial hemorrhagic bullae develop when minor trauma disrupts the epithelial-connective tissue interface, leading to bleeding from superficial capillaries. Lesions may be solitary or multiple and are most often reported in middle-aged individuals [11]. Although the soft palate is the predominant site, other regions such as the pharyngeal wall, esophagus, anterior faucial pillars, epiglottis, arytenoids, buccal mucosa, and the lateral and ventral tongue margins may also be affected [2,8]. Clinically, patients usually present with a hemorrhagic bulla that ruptures to form an erosion or ulcer, which generally heals spontaneously within days. Symptoms are typically mild, causing a foreign body sensation or sometimes a burning or tingling sensation, although larger bullae may cause choking sensations and significant anxiety, occasionally prompting emergency consultations [2,12,13]. Raizada et al. also suggested a possible hormonal influence, describing ABH flares linked with the menstrual cycle, though no such correlation was observed in the present case [14].

Connective tissue disorders of the oral mucosa might lower the blood vessels' ability to anchor, resulting in hemorrhagic lesions following trauma that cause these pathologic diseases in patients [15]. It is important to differentiate this benign disorder's presentation from that of other, more dangerous conditions with similar presentations, such as Kaposi sarcoma or epidermolysis bullosa acquisita [16]. A thorough history, clinical examination, and basic investigations (complete blood count, coagulation profile, and blood glucose levels) are recommended. In selected cases, biopsy and direct immunofluorescence can aid diagnosis. In our case, routine blood investigations and coagulation workups were performed, but HbA1c, PT, and aPTT values were not available. Advanced imaging and histopathological biopsy were also not carried out, which we acknowledge as important limitations [8].

Diagnosis of ABH was provided based on the criteria proposed by Ordioni et al. Out of nine criteria, seven were positive in this case, including two of the main criteria (Table 2), which included clinically noticeable hemorrhagic bulla or erosion with a history of bleeding of the oral mucosa and exclusively oral or oropharyngeal localization, and five additional criteria, which included palatal localization, triggering event or promoting factor (food intake), favorable evolution without leaving a scar in a few days, tingling or burning sensation, and normal platelet count and coagulation profile [2].

**Table 2:** Criteria for diagnosing angina bullosa hemorrhagica (ABH). A positive ABH diagnosis requires fulfilling six of nine criteria, including criteria 1 and 2, which are compulsory. Seven of the nine diagnostic criteria for ABH outlined by Ordioni et al. were fulfilled in this case. Adapted from: Ordioni et al. (2019) [2].

Criterion	Indication
Lesions in the mouth	Oral mucosal hemorrhagic bullae or erosions with a bleeding history
Location	Oral/oropharyngeal alone
Most common site	Palate
Triggering factors	Typically, food consumption or another motivating factor
Recurrence	Over time, lesions may resurface
Recovery	No scars, resolves in a few days
Sensation	Usually mild, but may tingle or burn
Laboratory investigations	Normal coagulation and platelet profiles
Immunological results	Negative direct immunofluorescence

Nevertheless, in situations where a biopsy is performed, microscopic analysis reveals a blood-filled subepithelial bulla and an underlying, mild, nonspecific mononuclear inflammatory cell infiltrate that is often restricted to the lamina propria region. Neutrophils can occasionally be observed [17].

Reassuring the patient is necessary for the management of the lesion. The goal of symptomatic treatment is often to improve ulcer healing and alleviate discomfort resulting from blister disruption. Any big bulla that has the potential to restrict airways should be surgically managed to avoid the obstruction [18].

In the present case, corticosteroids and topical antimicrobials were prescribed. While not routinely recommended for ABH, their use reflected the clinician’s attempt to address possible differential diagnoses. This represents another limitation of our report, as the therapy deviated from standard conservative management. Nonetheless, the lesions resolved completely without any complications.

Overall, ABH carries an excellent prognosis. Recognition of its benign, self-limiting nature is essential to avoid unnecessary interventions and to reassure patients appropriately.

## Conclusions

ABH of the oral cavity is an uncommon but benign condition that typically presents as sudden blood-filled blisters, most often on the soft palate. Although these lesions rupture spontaneously and heal within days, their sudden onset may cause patient concern, and a lack of awareness among practitioners may lead to unnecessary interventions. Diagnosis is primarily clinical and requires careful exclusion of other blood-filled bullous disorders through history, examination, and appropriate investigations. While the present case was diagnosed clinically, additional investigations such as coagulation studies, HbA1c, and biopsy may further strengthen diagnostic precision and were acknowledged as limitations. Increasing awareness of ABH among clinicians is essential for early recognition, accurate diagnosis, avoidance of unnecessary therapy, and appropriate patient reassurance.

Clinical takeaway from this report is that ABH should be considered in middle-aged patients presenting with sudden palatal hemorrhagic bullae to prevent misdiagnosis and overtreatment.
